# Understanding the Workflows in Dynamic and Robotic Computer‐Assisted Implant Surgery

**DOI:** 10.1002/cre2.70419

**Published:** 2026-07-17

**Authors:** Adria Jorba‐Garcia, Shengchi Fan, Feng Wang, James Chow, Atiphan Pimkhaokham, Nikos Mattheos, Alessandro Pozzi

**Affiliations:** ^1^ Department of Oral Surgery and Implantology, Faculty of Medicine and Health Sciences University of Barcelona Barcelona Spain; ^2^ Implants and Esthetics, Faculty of Dentistry Chulalongkorn University Bangkok Thailand; ^3^ Division of Oral and Maxillofacial Surgery, Faculty of Dentistry The University of Hong Kong SAR Hong Kong China; ^4^ Brånemark Osseointegration Centre Hong Kong China; ^5^ Oral and Maxillofacial Surgery and Digital Implant Surgery Research Unit, Faculty of Dentistry Chulalongkorn University Bangkok Thailand; ^6^ Department of Dental Medicine Karolinska Institute Stockholm Sweden; ^7^ Periodontology and Implant Dentistry The University of Hong Kong Hong Kong China; ^8^ Department of Clinical Science and Translational Medicine University of Rome Tor Vergata Rome Italy; ^9^ Department of Restorative Sciences Augusta University Augusta Georgia USA; ^10^ Department of Restorative Dentistry and Biomaterials Sciences Harvard School of Dental Medicine Boston Massachusetts USA

## Abstract

Computer‐assisted implant surgery (CAIS) represents a comprehensive digital workflow integrating prosthetically driven planning with various execution modalities. Among these, dynamic (d‐CAIS) and robotic (r‐CAIS) systems constitute two distinct yet interrelated approaches, sharing core technological principles as well as operative differences. This white paper aims to provide a structured overview of the fundamental concepts and workflows underpinning d‐ and r‐CAIS, focusing on their shared components, procedural steps, and system‐specific characteristics. Both approaches rely on accurate digital treatment planning, spatial positioning systems, registration protocols, and real‐time feedback mechanisms to guide implant placement. However, while d‐CAIS enables operator‐controlled freehand execution under real‐time navigation, r‐CAIS introduces robotic assistance or task autonomy, integrating mechanical actuation into the surgical workflow. Rather than providing exhaustive technical instructions, this work aims to establish a conceptual framework to support clinicians in understanding and evaluating emerging CAIS technologies and their role in contemporary implant dentistry. Furthermore, it discusses current advantages, limitations, and challenges related to clinical implementation, cost, and training requirements.

**Abbreviations**
3D: three‐dimensional
AI: artificial intelligence
AR: augmented reality
CAD‐IPS: computer‐aided design implant planning software
CAIS: computer‐assisted implant surgery
CBCT: cone‐beam computed tomography
CPU: central processing unit
d‐CAIS: dynamic computer‐assisted implant surgery
IOS: intraoral scanner/intraoral scanning
ITI: International team for implantology
ng‐CAIS: non‐guided computer‐assisted implant surgery
r‐CAIS: robotic computer‐assisted implant surgery
STL: standard tessellation language
s‐CAIS: static computer‐assisted implant surgery

## 1 Introduction

Computer‐assisted implant surgery (CAIS) is a modern comprehensive workflow in Implant Dentistry, with the Digital Treatment Plan as an essential prerequisite. Once the prosthetically driven treatment plan is finalized, the workflow of the implant placement can be executed with four approaches: non‐guided (ng‐), static (s‐), dynamic (d‐), and robotic (r‐) CAIS (Jorba‐Garcia et al. 2025).

Non‐guided CAIS protocols were the first to be developed, aiming to assist prosthetically driven implant placement by analog visual aids (Jorba‐Garcia et al. 2026), while later s‐CAIS introduced digitally manufactured, precise surgical guides and became the established standard thanks to widely documented accuracy (Yeo et al. 2025a; Tahmaseb et al. 2018).

Dynamic CAIS, on the other hand, is a totally different paradigm, based on optical tracking guidance, a technology originally developed for neurosurgery (Kaduk et al. 2013). By continuously tracking the position of surgical instruments relative to the patient's anatomy, d‐CAIS enables freehand surgery, while offering real‐time intraoperative visualization and adjustment of the instruments (Block and Emery 2016). After a period of experimentation and constant improvement, d‐CAIS technology is today widely documented as a reliable and accurate tool for implant placement, while several commercially available devices are competing to enter mainstream implant practice.

In the continuum of digital implant surgery workflows, r‐CAIS is the latest promising addition (Wu et al. 2019; Liu et al. 2024; Zhou et al. 2024). Rather than an entirely new technological paradigm, r‐CAIS is an expansion of dynamic navigation systems to the realm of collaborative or autonomous robotics (Tuygunov et al. 2026). As such, the digital treatment plan remains at the core, but instead of the surgeon's hand, the tracking system guides the end effector attached to a robotic arm. Although not yet anywhere close to reaching mainstream implant practice, r‐CAIS is already beyond the proof of principle, with thousands of documented cases and dozens of clinical trials already presenting unprecedented trueness of implant placement at the meta‐analysis level (Tuygunov et al. 2026).

The aim of this ITI white paper is to provide a comprehensive overview of the fundamental principles and concepts of d‐ and r‐CAIS across the different devices and protocols, as well as the essential shared steps of the workflow. As such, this paper is intended to provide structured orientation rather than exhaustive technical instruction and to introduce clinicians to key concepts, which will be further explored in more specific works. The readers are advised to navigate the presented workflows with the support of the Glossary of CAIS and related terms (Jorba‐Garcia et al. 2025), which is an essential complement to the content in this paper. Finally, this white paper aims to offer a comprehensive overview of the potential and limitations, cost‐related factors, as well as indications/contraindications and other parameters relevant to clinical decision making.

## 2 Preoperative Considerations

### 2.1 The Digital Treatment Plan and the Dynamic/Robotic Workflow

**Figure 1:**
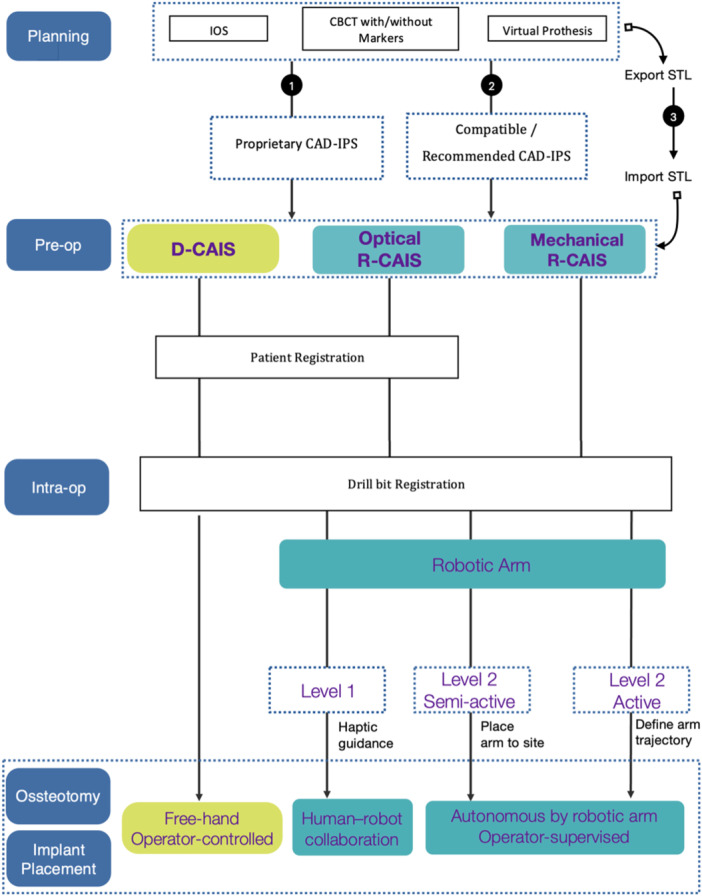
An overview of the workflow with dynamic and robotic CAIS, starting with the digital treatment plan (Planning), which is thereafter sent to execution to the d‐ or r‐CAIS device by means of three pathways (1:Proprietary, 2:Compatible CAD‐IPS, or 3:Export‐import STL). Execution of pre‐operative and intra‐operative procedures thereafter follows three vertical pathways for d‐CAIS (left), optical tracking r‐CAIS (middle), and mechanical tracking r‐CAIS (right). Finally, three tracks are defined for the osteotomy and implant placement, depending on the autonomy level of the r‐CAIS system.

As with all CAIS workflows, the digital treatment plan remains an essential prerequisite of working with either dynamic or robotic pathways (Jorba‐Garcia et al. 2026). However, the choice of the computer‐aided design—implant planning software (CAD‐IPS) could have significant implications for the workflow in d‐ and r‐CAIS, due to limitations with interoperability of different software and devices (Figure 1). Furthermore, some CAIS systems require certain provisions already at the stage of cone beam computed tomography (CBCT) acquisition. For instance, certain dynamic and robotic systems based on optical tracking technologies may require CBCT imaging to be performed with a radiopaque fiducial marker or clip in place to enable subsequent patient registration prior to surgery. Other systems are designed to utilize any CBCT, while the respective patient tracker is designed in the CAD‐IPS during the digital treatment plan (Tables 1 and 2).

**Table 1:** Overview of common dynamic navigation CAIS systems and respective technical features and specifications.

System/Launch year	Manufacturer/Region approved	Camera/Tracking system	Marker type	CAD‐IPS compatibility	Registration	Tracker reference location	Instrument registration	Display type	AI/Automation features
X‐Guide, 2015	X‐Nav Technologies/USA (FDA), CE	Pole‐mounted stereoscopic cameras/Infrared optical tracking	Passive optical markers	DTX import STL DICOM compatible	X‐clip marker, tooth tracing, surface‐based registration	Intraoral tooth/bone‐mounted tracker	Manual drill selection + registration plate/TCP calibration	External chairside monitor	ML‐assisted segmentation in recent software iterations
Navident, 2014	ClaroNav/Canada, CE, FDA‐cleared regions	Stereo visible‐light cameras/optical tracking	Passive optical markers	Proprietary; import STL DICOM compatible	Trace registration, fiducial clip, bone fiducial	Intraoral or extraoral, depending on workflow	Manual drill library + automated geometric calibration	External chairside monitor	Workflow‐guided registration automation
Dcarer, 2023	Dcarer Medical/China (NMPA), CE	Pole‐mounted stereoscopic cameras/Infrared optical tracking	Optical tracking with AI‐assisted surface recognition	Proprietary; import STL DICOM compatible	AI‐assisted markerless surface registration; optional optical markers	Intraoral tooth/bone‐mounted tracker	Registration plate/TCP calibration	External chairside monitor	AI‐powered surface recognition and multimodal fusion
Falcon, 2024	Straumann/CE	Handpiece‐mounted camera/Visible‐light optical tracking	Passive optical markers	coDiagnostiX	Digital marker‐based registration	Intraoral tooth/bone‐mounted tracker	Registration plate/TCP calibration	External chairside monitor/head‐up display	
Yake Navigation Platform, 2021	Yakebot Technology/China (NMPA), CE	Integrated optical cameras/Infrared tracking		Proprietary	Fiducial‐based registration	Intraoral tooth/bone‐mounted tracker		External chairside monitor	
IRIS Navigation, 2020	IRIS/CE	Pole‐mounted stereoscopic cameras/Infrared optical tracking		Proprietary import STL DICOM compatible	Fiducial and tooth‐supported registration	Intraoral tooth/bone‐mounted tracker	Registration plate/TCP calibration	External chairside monitor	

Abbreviations: CAD‐IPS = computer‐aided design implant planning software, CE = Conformité Européenne (EU), FDA = Food and Drug Administration (USA), ML = machine learning, NMPA = National Medical Products Administration (CN), TCP = tool center point.

**Table 2:** Overview of common robotic CAIS systems and respective technical features and specifications.

System/Manufacturer, year	Region approved	Autonomy level	Spatial positioning system	Tracking/Registration method	CAD‐IPS compatibility	Instrument calibration	Optical tracking dependency
Yomi/Neocis, 2017	USA (FDA)	Level 1	Mechanical arm positioning	Intraoral splint‐based mechanical registration	Proprietary	TCP calibration	No
Remebot/Baihui Weikang, 2021	China (NMPA)	Level 2—Semi‐active	Optical + robotic arm	Optical/fiducial	Proprietary	TCP calibration	Yes
Yakebot/Yakebot Technology, 2021	China (NMPA), EU (CE‐marked under EU MDR)	Level 2—Active	Optical + robotic arm	Optical/fiducial	Proprietary	Automated calibration	Yes
Dcarer/Dcarer Medical, 2023	China (NMPA)	Level 1	Optical + robotic arm	Optical/AI‐assisted surface registration or fiducial	Proprietary	Drill registration	Yes
THETA/Hangzhou Jianjia, 2023	China (NMPA)	Level 2—Semi‐active	Optical + robotic arm	Optical/fiducial	Proprietary	TCP calibration	Yes
Langyue (Cobot)/Shecheng, 2021	China (NMPA)	Level 2—Semi‐active	Mechanical + collaborative arm	Optical/fiducial	Proprietary	Mechanical calibration	Reduced
Human‐robot collaborative (HRCDIS)/Universal Robots, 2022	None specified	Level 2—Semi‐ative	Mechanical + collaborative arm	Optical/fiducial	Research workflow	TCP calibration	Partial
Hybrid robotic (HRSDIS)/Research prototype, 2023	None specified	Level 2—Semi‐active	Hybrid optical‐mechanical positioning	Optical/fiducial	Research workflow	Automated TCP calibration	Yes
X‐nav 2027 (anticipated)	X‐Nav Technologies (USA)	Level 1	Optical + robotic arm	Optical/fiducial	DTX	TCP calibration	Yes
Plan T—Aidite (2025)	Plan T, Yangshan Medical—Aidite, China	Level 1	Mechanical + collaborative arm	Intraoral splint‐based mechanical registration	Proprietary	TCP calibration	No

Abbreviations: CAD‐IPS = computer‐aided design implant planning software, CE = Conformité Européenne (EU), FDA = Food and Drug Administration (USA), NMPA = National Medical Products Administration (CN), TCP = tool center point.

In general, implementation of the digital treatment plan in the actual workflow follows three alternative pathways:

a. Using a “built‐in” CAD‐IPS:
  Most d‐ and r‐CAIS systems will come with their own proprietary CAD‐IPS, which allows the placement of the virtual implant based on the patient's CBCT. Most such systems, however, lack detailed design functionality of the prosthesis, thus prioritizing a bone‐driven placement. At the same time, they might lack significant details of the selected implant design features and connection, often using generic cylindrical “placeholders” instead of the actual virtual implant (Figure 1).
b. Using a compatible/recommended CAD‐IPS:
  Some d‐ and r‐CAIS systems are compatible with major commercially available CAD‐IPS, thus allowing the planning to take place in a more powerful software platform and thereafter to be seamlessly exported to the d‐CAIS system. This would allow the transfer of important details to the navigation system, such as exact implant shape or internal connection orientation. Compatibility is, however, often limited to each vendor's ecosystem, which limits the ability of the operator to freely choose implant system, software, and hardware. Using the recommended CAD‐IPS might be essential in the case where the patient marker (e.g., Falcon navigation, Straumann AG, Basel, Switzerland) (Werny et al. 2025) or a customized registration device (e.g., Yakebot, Yake Medical, Beijing, China) is digitally designed in the CAD‐IPS during the treatment planning phase.
c. Export–Import:
  In cases where there is no interoperability between the preferred CAD‐IPS and the d‐CAIS system, operators can choose to export the patient‐optimized implant position from the software used for digital treatment plan (e.g., Exoplan, Exocad, Germany) as an STL file and thereafter import it into the d‐CAIS proprietary system. Apart from potential loss in accuracy, this process often results in losing important information, such as the prosthesis design, the exact shape of the implant, or the orientation of the implant connection. This limitation reflects the lack of standardized data formats for fully preserving implant‐ and prosthesis‐related information across platforms (Figure 1).

### 2.2 Essential Components of a Dynamic CAIS/Navigation System

**Figure 2:**
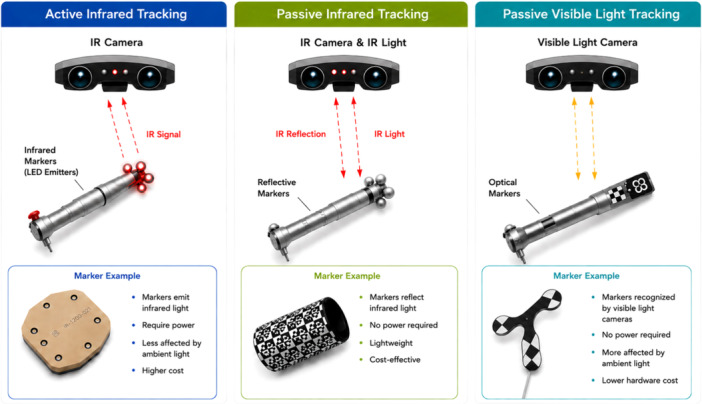
Schematic comparison of optical tracking technologies used in CAIS. Active infrared tracking is based on markers equipped with infrared emitters, whereas passive infrared tracking uses reflective markers illuminated by an external infrared light source. Passive visible‐light tracking employs passive optical markers detected by visible‐light cameras through image‐processing algorithms.

**Figure 3:**
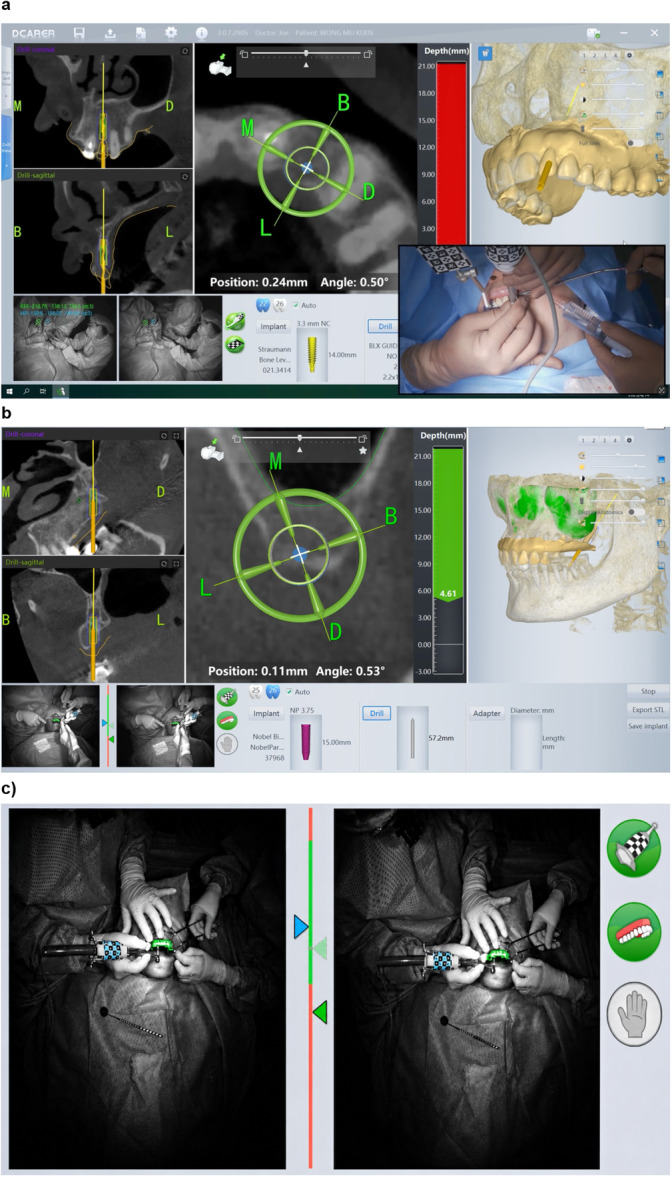
Screenshot of the operator's view of the dynamic‐CAIS system. Among the commonly used features, note the directional deviation marker (target shape, centre) and depth indicator (red vertical bar, centre). (a) Optical tracking maintained through a patient tracker attached to the maxilla with an intraoral splint, with the markers at the handle of the handpiece. Note in the Intraoperative view (lower right window) the two‐hand grip of the operator, typical for dynamic navigation surgery (DCarer, China. 4th generation). (b) Dynamic‐CAIS system without optical patient markers. Note the camera field view (lower left) where the green tracking area corresponds to the patient's anterior teeth and the blue tracking area at the markers of the handle of the handpiece (c) (DCarer, China. 6th generation).

A d‐CAIS system is based on three core technologies: (a) spatial positioning system, (b) display system, and (c) control system, designed to provide real‐time guidance of surgical instruments used for osteotomies, implant site preparation, and implant placement (Jorba‐Garcia et al. 2025).

a. The spatial positioning system allows the control system to track the movements of the surgical instruments in real time. Most contemporary d‐CAIS systems rely on optical tracking technology for this purpose (Block and Emery 2016). Optical markers are rigidly attached to the surgical handpiece and the patient, which remain within the line of sight of stereoscopic cameras. The cameras may operate in either visible or infrared light, while the tracking mechanism can be categorized as active or passive, based on active light emission or not by the markers/trackers (Figure 2). Finally, the cameras are mounted on a special arm for positioning in front or above the patient, or can be mounted on the contra‐angle handpiece.
b. The display system is the visualization instrument during the surgery, as it projects in real time the position of the surgical instruments on the patient's anatomic representation, while it also provides information with regard to the angular and linear deviation from the planned position (Jorba‐García et al. 2021). This visualization is typically projected on a computer screen or a head‐up display (Figure 3a,b) (Arunjaroensuk et al. 2024).
c. The control system is a dedicated computer Central Processing Unit (CPU), which receives input from the optical tracking system and provides output to the display system after processing (Block and Emery 2016). This system creates a virtual space directed by 3D coordinates and registers the position of the patient's jaw and the surgical instruments against these common coordinates. Thereafter, receiving data continuously from the spatial positioning system, it monitors the relative position of the instruments and the patient's jaw in the common coordinate frame during the surgery, using triangulation algorithms.

### 2.3 Essential Components of a Robotic CAIS System

**Figure 4:**
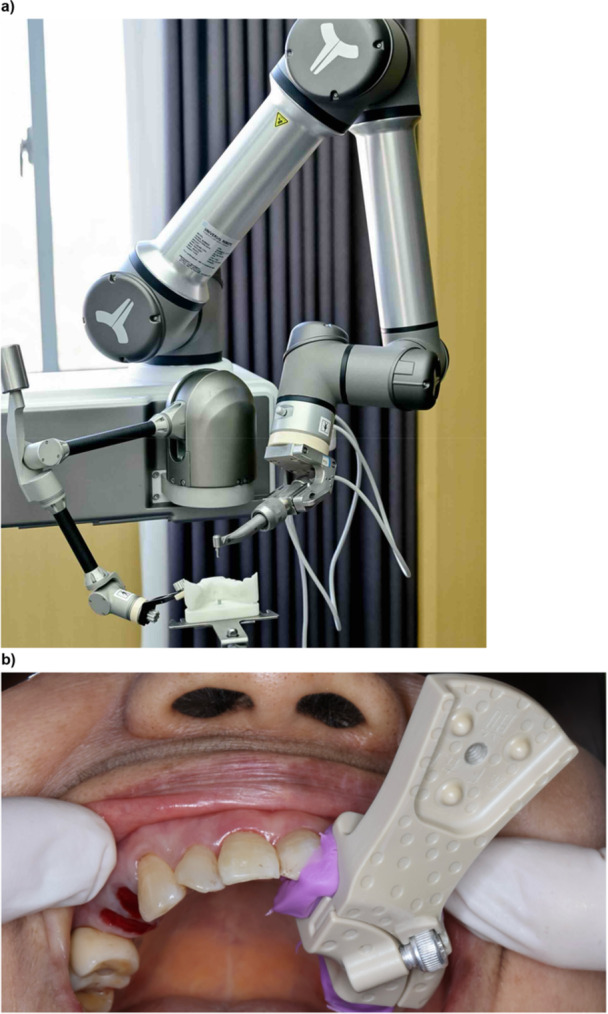
A collaborative CAIS robot with a mechanical tracking arm in vitro operational display (Plan T, Yangshan Medical/Aidite, China). (a) Note the mechanical spatial positioning arm attached firmly to the jaw by means of a custom‐made intraoral splint (left—small arm), while the robotic arm (right—big arm) restricts the movement of the end effector. (b) Intraoral splint with a clip for the attachment of the mechanical positioning arm.

As r‐CAIS is an extension of the dynamic navigation technology in the domain of robotics, CAIS robots share many core components and functions with navigation systems, unless they use mechanical or haptic positioning, which uses different technologies. Consequently, most contemporary r‐CAIS systems incorporate navigation functionality as an integral part of their workflow. Similar to CAIS navigation, CAIS robots also comprise: (a) a spatial positioning system, (b) a display system, and (c) a control system. In addition to these, r‐CAIS systems also incorporate (d) a robotic arm with an end‐effector, and (e) sensing module (Wang et al. 2025).

a. The spatial positioning system. Similar to navigation surgery, optical positioning systems are the most common in CAIS robots (Chen et al. 2023, 2024; Bolding and Reebye 2022; Dibart et al. 2023; Zhuang et al. 2024; Yang et al. 2024). Some robots, however, rely on mechanical positioning (e.g., Yomi, Neocis Inc., Miami, USA), which represents one of the earliest principles applied in robotic‐assisted surgery (Wu et al. 2019). In this approach, spatial positioning is achieved through an additional mechanical arm, firmly attached to an intraoral splint (Figure 4). This allows for continuous tracking of the relative position of the drills and the patient, without the need for cameras and optical trackers in the line of sight. Nevertheless, the accuracy of mechanical tracking can be affected by manufacturing tolerance and mechanical wear, making external calibration and verification essential to ensure consistency between planned and actual instrument positions.
b. The display system. Similar to navigation surgery, CAIS robots utilize computer screens to display the position of the surgical instruments on the patient's anatomic representation in real time. Thanks to the sensing module, however, displays can provide more intraoperative information, such as the presence and direction of bone resistance during the drilling, the forces applied from the socket walls, and more.
c. The control system. Similar to navigation surgery, the control system receives input from the spatial positioning system and provides output to the display system after processing. Its primary function, however, is to control the robotic arm and the end‐effector toward preparing the osteotomy and placing the implant as directed by the digital treatment plan and the 3D coordinates. In addition, it receives input from the surgeon through a foot pedal and/or handheld controllers, who, in task autonomous systems, must also define the motion trajectory of the robotic arm.
d. The robotic arm is an essential part of the CAIS robot, and it consists of an articulated robotic arm (actuator), which controls a handpiece (end effector) and enables the execution of the osteotomy and implant placement according to the planned trajectory. Robotic arms can vary considerably in their design characteristics, with the number of joints determining the degrees of freedom of movement and the flexibility of the arm to position the end effector accurately in the three‐dimensional (3D) space.
e. The sensory module consists of several hardware and software components in different parts of the robot (e.g., force feedback or tactile sensors). By monitoring the magnitude and direction of forces applied to the end‐effector, the system can infer bone hardness, evaluate resistance encountered during drilling, and provide feedback to improve surgical accuracy and safety.

### 2.4 Robotic Autonomy

The extent and nature of the tasks that are executed by the robotic arm determine the level of autonomy. Yang et al. proposed a classification framework for medical robots based on levels of autonomy, ranging from Level 0 to Level 5, with progressively increasing degrees of system independence. Within this framework, currently available r‐CAIS systems are primarily categorized as Level 1 (collaborative or passive) and Level 2 (task‐autonomous) (Yang et al. 2017).

Level 1 systems require continuous manual control by the clinician throughout the procedure. In these systems, the robotic platform provides mechanical guidance, stabilization, and constrains the motion, while the surgeon physically holds the handpiece during implant site preparation and placement.

In contrast, Level 2 task‐autonomous systems can autonomously execute predefined tasks—such as osteotomy preparation or implant placement—according to the preoperative plan, while remaining under continuous clinical supervision. Level 2 systems may be further subdivided into semi‐active, in which the surgeon positions the robotic arm intraorally before automated task execution, and active, which are capable of autonomously reaching the surgical site and completing implant bed preparation once the trajectory of the entry and exit movement is defined.

## 3 Common Pre‐Operative Workflow Steps in Dynamic and Robotic CAIS

### 3.1 Patient and Instrument Registration

Registration is a critical step in both d‐ and r‐CAIS systems that rely on optical tracking for spatial positioning (Jorba‐Garcia et al. 2025). This step ensures that the position of the patient, the instruments, and the digital treatment plan are aligned with a common 3D coordinate system. The control system replicates the physical space to an exact virtual one and empowers the real‐time exact virtual alignment of the 3D CBCT data, the digital treatment plan with the planned implant position, and the patient's actual anatomy. Thus, once this virtual space is created, registration of (a) the handpiece, (b) all respective drill bits and instruments, and (c) the patient will ensure accuracy of the procedure. The patient tracker needs to be firmly attached to the patient's jaw prior to registration, with the exception of one recently launched d‐CAIS system, which can identify the position of the patient by optically tracking the anterior teeth.

#### 3.1.1 Handpiece and Instruments/Drill Bit Registration

Registration of the handpiece and each drill bit in the patient and data 3D coordinate systems is the essential final step before commencing the surgery. Clinicians must register the handpiece first through the drill axis or orientation. This step is performed by attaching a registration device to the handpiece socket for drill bits and placing it in the line of sight of the camera. As most optical tracking systems do not directly measure the length of each drill bit, the effective drill type and length are defined through an additional registration process. There are mainly two ways for achieving accurate drill bit/instrument registration:

1. Manual selection of pre‐registered drills: The d‐ or r‐CAIS system maintains a library of drill types and dimensions, and manual selection of each drill bit is conducted by the operator.

2. Registration plate: The operator mounts the respective drill to the handpiece and positions it on a specially made registration plate in the line of sight of the stereoscopic cameras.

#### 3.1.2 Patient Registration for Systems With Optical Tracking

**Figure 5:**
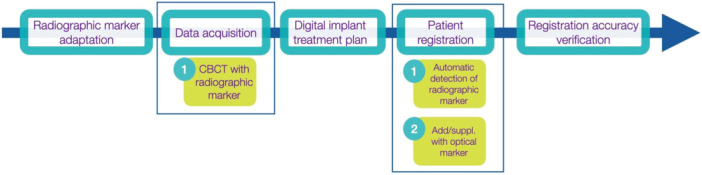
Workflow of radiographic marker‐based registration in CAIS. A radiographic marker is first adapted to the patient and incorporated into the CBCT acquisition process. The acquired dataset is subsequently used for digital implant treatment planning. During patient registration, the radiographic marker is automatically detected and matched to its corresponding physical position, thereby establishing the spatial relationship between the patient and the virtual treatment plan. In some systems, optical markers may be added to supplement tracking and facilitate registration. Finally, registration accuracy is verified before surgery to ensure accurate transfer of the planned implant position to the clinical environment.

**Figure 6:**
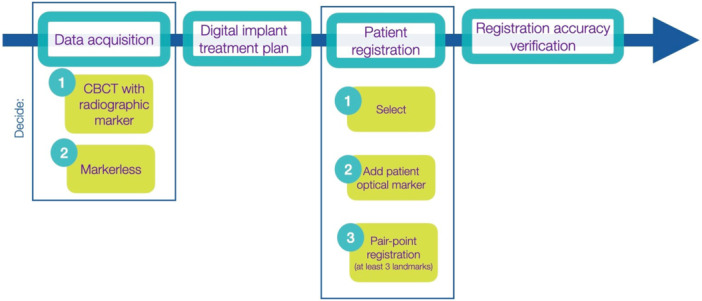
Workflow of pair‐point registration in CAIS. Following CBCT acquisition and digital implant planning, a patient tracker is attached to enable optical tracking. Pair‐point registration is subsequently performed by identifying and matching at least three corresponding anatomical landmarks on the patient and within the CBCT dataset. This process establishes a common coordinate system between the patient and the virtual treatment plan. Registration accuracy is then verified before surgery to ensure precise navigation throughout implant placement.

Two main patient registration procedures are commonly utilized in d‐ and r‐CAIS systems, which utilize optical tracking technology:

1. Radiographic, marker‐based registration (see term D17 (Jorba‐Garcia et al. 2025)), which involves a patient's radiopaque marker, typically by means of an intraoral splint or clip, which is worn during the CBCT acquisition (Figure 5).
2. Pair‐point registration (see term D18 (Jorba‐Garcia et al. 2025)), which is based on selecting fiducial points or anatomical landmarks on the CBCT images (most commonly cusps, incisal edges, or other stable dental structures) and subsequently tracing the corresponding locations in the patient's mouth using a calibrated probe equipped with optical markers. Depending on the fiducial reference used, pair‐point registration can be further classified into (a) markerless and (b) radiographic marker pair‐point registration (e.g., utilizing mini‐screws, adhesive radiopaque markers, or dedicated devices) (Figure 6).

Other common procedures for patient registration include:

− Point‐based and surface‐enhanced registration (see term D19 (Jorba‐Garcia et al. 2025)), where point‐based matching can be supplemented with surface mapping of the surrounding anatomical area, aiming to enhance registration robustness and overall accuracy.
− Digital marker‐based registration (see term D20 (Jorba‐Garcia et al. 2025)), where a patient‐specific tray or template is digitally designed during the digital treatment plan and subsequently manufactured with additive manufacturing techniques. During surgery, the optical marker is mounted onto the pre‐fabricated template and is immediately recognized by the tracking system, obviating the need for additional intraoperative registration steps. (Falcon, Straumann AG, Basel, Switzerland) (Werny et al. 2025)
− Artificial intelligence (AI)‐powered, trackerless registration, where AI‐powered image processing enables the navigation tracking system to detect the surface of the teeth intraoperatively and automatically align the patient's jaw with the CBCT volume and intra‐oral scanning (DCarer 6th Generation, China) (Wu et al. 2025a). In certain systems, the application of temporary optical reference markers, such as colored resin dots bonded to the buccal surfaces of the teeth, may be required to enhance surface recognition and tracking robustness (Ma et al. 2022).

In fully edentulous patients, d‐ and r‐CAIS systems require registration strategies that compensate for the absence of tooth‐supported reference points. Registration in such cases is typically achieved using bone‐anchored fiducial devices or customized surgical templates rigidly fixed with miniscrews, often supplemented by intraoperative CBCT to confirm registration accuracy.

#### 3.1.3 Patient Registration for Systems With Mechanical Tracking

Mechanical spatial positioning robotic systems follow a different mode of spatial alignment, which does not require the same registration process as in the optical tracking systems. In these systems, an intraoral stent is incorporated into the preoperative imaging and planning phase, establishing the spatial relationship between the patient's anatomy and the stent. Intraoperatively, the robotic system localizes this stent through mechanical coupling with a separate tracking arm, which effectively preserves this relationship without the need for a separate image‐to‐patient registration step. As a result, spatial correspondence between the tracking system and the robotic arm is inherently preserved, and no separate patient registration procedure is required.

### 3.2 Calibration of Robotic and Dynamic Systems

Calibration is the comparison of measurement values delivered by a device under test with those of a standard of known accuracy. Calibration is an entirely different process from registration and should not be confused (Jorba‐Garcia et al. 2025), although in the past the two terms were at times used interchangeably. All devices within CAIS workflows (CBCT, IOS, CAD‐IPS, etc.) require calibration to ensure performance at industry standards. This process is, however, not required for every patient and is often performed by manufacturers or certified experts.

Optical tracking robotic systems, in particular, require calibration to establish an accurate spatial relationship between the camera system and the robotic arm. This is typically performed using standardized calibration objects—such as checkerboard grids or symmetric and asymmetric circular patterns with known geometric dimensions—which are temporarily mounted on the robotic arm. System‐guided movements are then executed while the camera acquires multiple positional datasets, from which transformation parameters are computed to enable accurate tracking and robotic motion control. Calibration of r‐CAIS systems is conducted regularly, but not essential before every surgery.

## 4 Intraoperative Workflow Steps

### 4.1 Dynamic CAIS

Following registration, the surgery with d‐CAIS involves the following steps:

1. Confirm stability of patient tracker: The stability and fixation of the patient tracking device must be verified prior to surgery and constantly monitored. Even minor displacement during the procedure may compromise navigation accuracy.
2. Verify the registration: The accuracy of registration between the patient and the virtual planning data should be confirmed before initiating the osteotomy. This is typically achieved by checking predefined anatomical landmarks to ensure consistency between the virtual and clinical environments.
3. Confirm unobstructed line of sight: A clear line of sight between the tracking cameras and the tracked objects must be maintained; the ergonomics of the surgery should be carefully planned. Any obstruction may interrupt tracking and compromise accuracy.
4. Confirm visual access and ergonomics: The operator should ensure direct and ergonomic visualization of the navigation display, allowing continuous real‐time feedback without compromising surgical posture or workflow efficiency.
5. Conduct the osteotomy: The osteotomy is performed in a freehand manner under real‐time navigation guidance, with the operator maintaining full control of the handpiece while continuously adjusting angulation and depth based on the displayed feedback.
6. Further instrument and drill bit registration: Each drill bit or instrument must be registered within the system to ensure accurate tracking of its position and orientation during use.
7. Placement of the implant: Implant insertion is carried out freehand under navigation guidance, with the operator controlling the trajectory, depth, and rotational positioning with real‐time feedback from the display system.

### 4.2 Robotic CAIS

**Figure 7:**
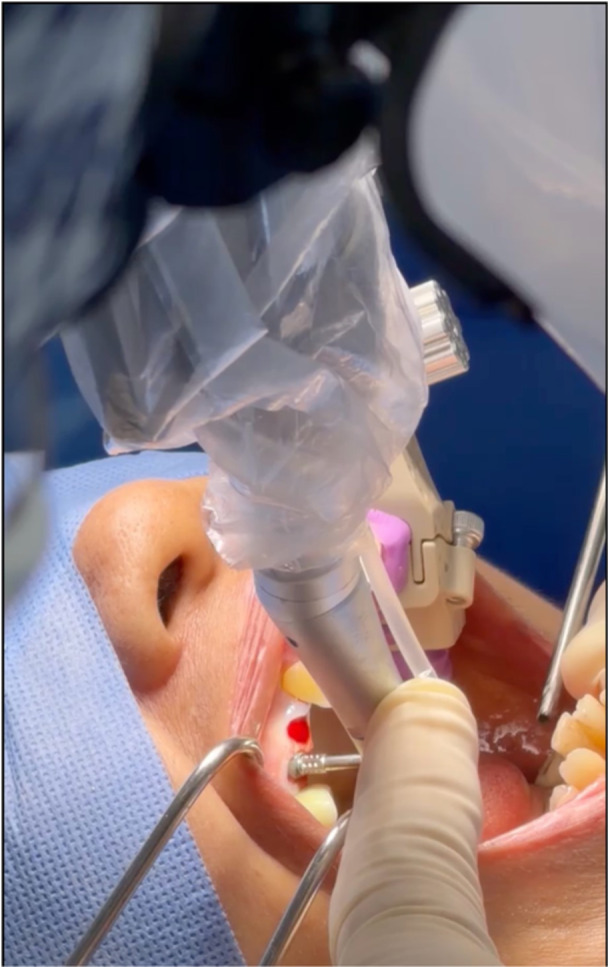
Implant placement with colaborative CAIS robot. The robotic arm is aligned with the planned trajectory at the final stage of placement and restricts the movement of the handpiece. The surgeon places the implant in the planned position by applying light finger pressure on the handpiece, within the range of movement allowed by the robotic arm (Plan T, Yangsan Medical/Aidite, China).

**Figure 8:**
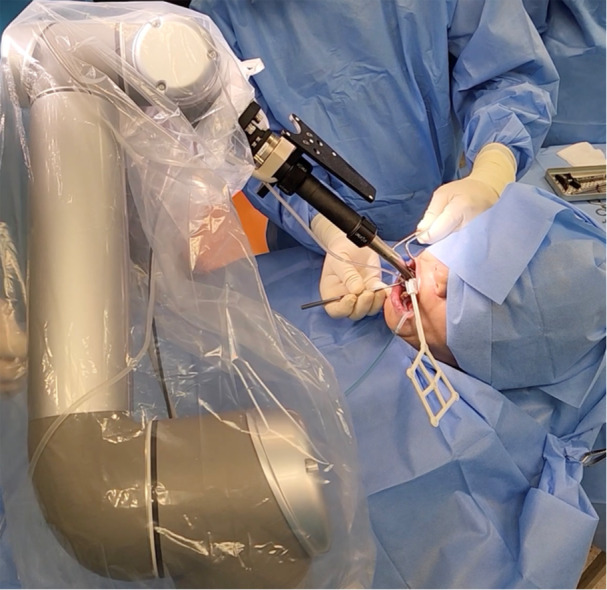
Implant placement with task autonomous CAIS robot. Note the optically guided robotic arm with the end effector autonomously conducting the osteotomy without the surgeon's guidance, who is, however, able to supervise the operation and interfere through a foot pedal. Optical tracking is possible through the patient tracker attached to an intraoral splint and the fiducial markers on the black plate attached to the end effector (Yakebot, Beijing Yakebot technology Co Ltd, China).

Workflow steps 1–4 as above in d‐CAIS repeated in the r‐CAIS as well. Then, in addition:

5. Position of the end effector intraorally (Yang et al. 2017):
  Level 1, autonomy: The surgeon holds the handpiece and moves it manually in the mouth, approaching the planned implant site.
  Level 2, semi‐active: The surgeon holds the handpiece and moves it manually in the mouth, approaching the planned implant site.
  Level 2, active: The surgeon defines the trajectory of motion by dragging the handpiece in and out of the mouth. The motion is registered, and thereafter the robotic arm repeats the movement to place and remove the handpiece.
6. Conduct the osteotomy:
  Level 1, autonomy: The robot initiates the drilling mode. The surgeon holds and moves the handpiece but only along the trajectory that is allowed by the robot, corresponding to the depth and angle of the planned position (Figure 7).
  Level 2, semi‐active or active: The robot initiates the drilling mode. The end effector moves autonomously to conduct the osteotomy, while the surgeon can supervise the process and interfere through a foot pedal or buttons (Figure 8).
7. Place the implant:
  Level 1, autonomy: The surgeon places the implant, holding and moving the handpiece along the trajectory allowed by the robot.
  Level 2, semi‐active or active: The end effector moves autonomously to place the implant while the surgeon can supervise the process and interfere through a foot pedal or buttons.

### 4.3 Modifications for Immediate Placement

The workflow for immediate implant placement using d‐CAIS and r‐CAIS does not differ substantially from that used in partially edentulous cases. In such a scenario, particularly when employing a markerless pair‐point registration approach, it may be beneficial to use the tooth planned for extraction as a landmark for registration prior to its extraction, as long as it has sufficient periodontal stability and does not present mobility. This allows for the selection of a reference point directly within the surgical area, which could enhance the robustness and accuracy of patient registration.

### 4.4 Modifications for Terminal Dentition

Teeth planned for extraction can be utilized to perform a markerless pair‐point registration only if they are stable and in sufficient number. In selected cases, strategically positioned teeth located outside the planned implant sites may be intentionally preserved until the completion of surgery to serve as stable support for optical tracking markers. Alternatively, additional artificial fiducial references will be required, such as bone‐anchored mini‐screws or other radiopaque fiducial.

### 4.5 Modifications for Edentulous Patients

Fully edentulous patients represent challenging scenarios in d‐CAIS and r‐CAIS, due to the absence of reliable anatomical landmarks, as osseous reference points alone may not provide sufficient registration accuracy.

Artificial fiducial references are commonly introduced to serve as landmarks for pair‐point registration. These may include bone‐anchored radiopaque markers, mini‐screws, or radiographic splints incorporating a minimum of three landmarks/fiducial markers. These artificial landmarks are traced intraoperatively prior to initiation of the drilling sequence. Other approaches, such as adhesive radiopaque markers or the exclusive use of osseous landmarks, have been reported; however, robust clinical evidence supporting their accuracy remains limited. Alternatively, particularly in the maxilla, extraoral optical tracking systems may be employed using headsets' reference frames supported at the nasion and auricular regions, thereby eliminating the need for intraoral fixation.

## 5 Discussion

Although both d‐ and r‐CAIS systems offer highly accurate implant placement and share several technological features, they constitute two distinct treatment modalities and workflows. Dynamic systems have been available for more than 20 years, thus having matured to efficient and predictable workflows. On the other hand, r‐CAIS systems are currently in infancy, despite having documented impressive accuracy of implant placement in multiple clinical scenarios. By introducing robotic platforms capable of physically assisting or autonomously executing surgical tasks, r‐CAIS integrates preoperative digital planning, spatial tracking, and robotic actuation into a unified system, which directly controls intra‐operative instrument motion. Since the first CAIS robot received regulatory clearance in 2017 (Yang et al. 2017), multiple platforms with varying positioning principles and levels of autonomy have been developed, reflecting a broader shift toward action‐oriented digital implant surgery (Table 2). Observing the rapid progress of robotics in other fields of surgery, one can only anticipate an increasing role of r‐CAIS systems in the near future.

### 5.1 Advantages and Limitations

d‐CAIS systems offer high accuracy (Pozzi et al. 2024, 2026), while presenting several advantages over the s‐CAIS alternatives. As the printing of a guide is not required, preoperative planning and surgical procedures can be performed on the same day. The absence of a surgical guide and the respective guided drills allows for easier implant placement in posterior sites or in patients with reduced mouth opening. It allows for intraoperative confirmation of accuracy at any time, reducing cumulative errors. Furthermore, as the surgery remains essentially freehand and under the full visual control of the operator, intraoperative changes are possible, while the full tactile perception alerts the surgeon in case of deficient bone density or other unexpected situations. This might often lead to higher implant primary stability of implants placed with navigation over those placed through surgical guides (Pimkhaokham et al. 2022), while it increases the educational value of d‐CAIS systems for the training of surgeons (Uei et al. 2025; Teparrukkul et al. 2024).

At the same time, d‐CAIS systems present their own drawbacks and limitations. Purchase of a d‐CAIS system at present requires a high initial investment, while some systems add incremental costs per use by requiring a license fee or single‐use patient stents. Although the user interface of d‐CAIS systems has remarkably improved, mastering their use requires significant commitment, structured training, and practice (Uei et al. 2025). The complexity of the componentry and registration procedures might prolong the duration of the surgery, disproportionally so in straightforward cases. Moreover, although intraoperative control of the accuracy is always possible, minor deviations due to registration errors or slight dislodgement of the patient tracker might go unnoticed.

The introduction of r‐CAIS initially aimed at reducing operator‐dependent variability and cumulative human error during complex procedures (Liu et al. 2024; Zhou et al. 2024; Bahrami et al. 2024). By translating virtual implant planning into mechanically constrained or robotically executed actions under continuous supervision, r‐CAIS has enhanced placement accuracy, procedural consistency, and reproducibility. Upon clinical assessment, the major advantage of r‐CAIS presented with was its incremental, yet clearly superior positional trueness compared with other CAIS approaches and non‐guided placement (Yang and Li 2024; Khan et al. 2024; Wu et al. 2024, 2025b; Pozzi et al. 2025; Luo et al. 2025; Schiavon et al. 2025; Jia et al. 2026a; Fan et al. 2026). Although clear from a statistical perspective, this increased level of trueness has not yet been shown to be clearly associated with superior clinical (Pimkhaokham et al. 2022) or patient‐reported outcomes (Yeo et al. 2025b). Thus, further research “beyond accuracy” (Sadilina et al. 2025) is required in order for robotic technologies to reach their full potential in implant dentistry.

Despite clear claims with regard to accuracy and consistency, currently available r‐CAIS systems come with significant constraints. Not only is the initial investment very high, but also high per‐patient costs persist, a reality that might challenge the return on investment for a clinic even in the long term. Further workflow‐related limitations can affect clinical scalability, as robotic implant workflows often involve additional preparatory steps, which increase procedural complexity and setup time. In a low‐complexity clinical scenario, such as single‐gap implant placement, this added workflow burden may outweigh the accuracy benefits and limit efficiency gains.

r‐CAIS remains resource‐intensive. Task autonomous robots, in particular, require specialized technical support, while procedures are often performed in the presence of manufacturer‐affiliated engineers. There appears to be a void with regard to the essential education and training pathways for clinicians, as the essential competences and skillsets to safely operate CAIS robots are currently not defined. Collectively, these factors highlight the need for further research and development in order to simplify the workflow, reduce acquisition and maintenance costs, define the essential skillsets and competence for the clinician‐operator, and further support clinical implementation for a wider scope of indications.

### 5.2 Cost Effectiveness and Related Factors

The cost‐effectiveness of all CAIS modalities is scarcely studied and typically only involves assessing isolated parameters such as the duration of the surgery. d‐CAIS is characterized by a high initial investment for acquisition of the system, but also a high cognitive investment in education and training, as both duration and performance are associated with operators' experience with the device. Thereafter, additional small incremental costs remain with some d‐CAIS systems, through single‐use devices such as thermoplastic stents or per‐patient license fees. The overall efficiency in clinical procedures might vary, with the benefits of d‐CAIS increasing with the complexity of the surgery and also the experience of the operator.

r‐CAIS, on the other hand, has primarily been assessed for trueness of implant positioning, with data supporting any conclusions on overall efficiency being extremely scarce. The high initial investment, the mostly under‐reported costs associated with consumables and additional human resources, as well as the inconsistent reporting on surgical duration, allow for no serious assessment of cost‐effectiveness and overall procedure efficiency of r‐CAIS systems at present.

### 5.3 Clinical Implementation

Although d‐CAIS systems have been available for about two decades already, clinical implementation in a modern practice is not without challenges. The lack of interoperability between major CAD‐IPS and the d‐CAIS systems is a pain point many clinicians experience when transitioning to d‐CAIS workflows. Most d‐CAIS systems come with their own proprietary CAD‐IPS, which are typically of limited functionality and do not allow a proper prosthetically driven treatment plan. At the same time, popular CAD‐IPS, which allow prosthetically driven treatment plans, are often compatible only with specific d‐CAIS systems, which greatly limits the ability of the operator to freely select implants, software, and d‐CAIS hardware. As a result, planning is often done in one platform and execution in another, with several export/import steps in between. This can be both a major burden and a significant limitation as important design information is lost in the process, while the risk for deviation and errors is increased.

Clinical implementation of r‐CAIS systems is far more challenging, with challenges from patient acquisition, to treatment planning platforms, physical space and ergonomics, training of operators, human and material resources for each clinical procedure, and more. Thus, although by now thousands of implants have been placed by r‐CAIS systems (Shuborna et al. 2026; Jia et al. 2026b), this has predominantly taken place in university hospitals or large specialist clinic environments. Miniaturization of the robotic technologies, simplification of the workflows, cost reduction, and increase in efficiency will be essential steps before robotic implant surgery can be further implemented in mainstream clinical practice.

### 5.4 Future Developments

Both d‐ and r‐CAIS are currently very active domains of research and development, with frequent technological breakthroughs. Competition is rising among industry partners, with multiple companies investing significantly in research and development, while research centers are being established in Universities and hospitals. Optimization of the technology can bring an incremental increase in efficiency, but occasional “game‐changers” such as tracker‐less optical spatial positioning promise to deliver better outcomes in a shorter time, while improving patient and operator experience.

From a workflow perspective, AI is expected to function as an enabling layer within future d‐ and r‐CAIS systems, supporting planning optimization, intra‐platform communication, intraoperative decision support, and outcome assessment. Within existing workflows, AI may serve as an adaptive computational component that interprets anatomical data, estimates bone quality, and defines safety boundaries relative to critical structures. With advances in sensing technologies, multimodal data acquisition, and computational control architectures, real‐time decision‐making support could be the next frontier. This could translate to higher levels of decision‐making for d‐CAIS systems or higher levels of robotic autonomy in robotic systems. In this context, Level 3 robotic systems may be envisioned as platforms capable of planning and executing complete implant procedures with dynamic intraoperative adjustment under continuous surgeon supervision. Rather than a transition to fully autonomous surgery, such systems would represent an incremental evolution of current r‐CAIS workflows, in which AI enhances execution robustness and consistency while clinical responsibility remains firmly with the operator.

## Author Contributions

Conceptualization: Adria Jorba‐Garcia, Sengchi Fan, Atiphan Pimkhaokham, Nikos Mattheos, Alessandro Pozzi. Methodology: James Chow, Atiphan Pimkhaokham, Nikos Mattheos, Alessandro Pozzi. Investigation: Adria Jorba‐Garcia, Shengchi Fan, Feng Wang. Resources: Feng Wang, James Chow, Alessandro Pozzi. Data curation: Nikos Mattheos, Alessandro Pozzi, James Chow. Project administration: Nikos Mattheos, James Chow. Supervision: Nikos Mattheos, James Chow, Alessandro Pozzi. Visualization: Shengchi Fan, Adria Jorba‐Garcia, James Chow. Writing – original draft: Adria Jorba‐Garcia, Shengchi Fan. Writing – review and editing: Sengchi Fan, Feng Wang, James Chow, Atiphan Pimkhaokham, Nikos Mattheos, Alessandro Pozzi. Funding acquisition: Nikos Mattheos, James Chow.

## Conflicts of Interest

The authors declare no conflicts of interest.

## Data Availability

Data sharing is not applicable to this article as no datasets were generated or analyzed during the current study.
